# Characterization of IncHI1B Plasmids Encoding Efflux Pump *TmexCD2-ToprJ2* in Carbapenem-Resistant *Klebsiella variicola*, *Klebsiella quasipneumoniae*, and *Klebsiella michiganensis* Strains

**DOI:** 10.3389/fmicb.2021.759208

**Published:** 2021-10-06

**Authors:** Yujiao Wang, Bo Zhu, Min Liu, Xiutao Dong, Jianping Ma, Xiaofeng Li, Fang Cheng, Jianzhuang Guo, Sumei Lu, Furong Wan, Yingying Hao, Wanshan Ma, Mingju Hao, Liang Chen

**Affiliations:** ^1^Department of Clinical Laboratory Medicine, The First Affiliated Hospital of Shandong First Medical University & Shandong Provincial Qianfoshan Hospital, Shandong Medicine and Health Key Laboratory of Laboratory Medicine, Jinan, China; ^2^Xiamen Key Laboratory of Genetic Testing, Department of Laboratory Medicine, The First Affiliated Hospital of Xiamen University, Xiamen, China; ^3^Department of Clinical Laboratory, Jinan Dermatosis Prevention and Control Hospital, Jinan, China; ^4^Department of Clinical Laboratory, Shandong Provincial Hospital Affiliated to Shandong First Medical University, Jinan, China; ^5^Center for Discovery and Innovation, Hackensack Meridian Health, Nutley, NJ, United States; ^6^Department of Medical Sciences, Hackensack Meridian School of Medicine, Nutley, NJ, United States

**Keywords:** *TmexCD2-ToprJ2*, carbapenem resistance, *bla*_NDM-1_, *bla*_NDM-5_, *Klebsiella variicola*, *Klebsiella quasipneumoniae*, *Klebsiella michiganensis*, tigecycline resistance

## Abstract

Tigecycline serves as one of the last-resort antibiotics to treat severe infections caused by carbapenem-resistant Enterobacterales. Recently, a novel plasmid-mediated resistance-nodulation-division (RND)-type efflux pump gene cluster, *TmexCD1-ToprJ1*, and its variants, *TmexCD2-ToprJ2* and *TmexCD3-ToprJ3*, encoding tetracyclines and tigecycline resistance, were revealed. In this study, we reported three *TmexCD2-ToprJ2*-harboring *Klebsiella* species strains, collected from two teaching tertiary hospitals in China, including one *K. quasipneumoniae*, one *K. variicola*, and one *K. michiganensis*. The three strains were characterized by antimicrobial susceptibility testing (AST), conjugation assay, WGS, and bioinformatics analysis. AST showed that *K. variicola* and *K. quasipneumoniae* strains were resistant to tigecycline with MIC values of 4μg/ml, whereas the *K. michiganensis* was susceptible to tigecycline with an MIC value of 1μg/ml. The *TmexCD2-ToprJ2* clusters were located on three similar IncHI1B plasmids, of which two co-harbored the metallo-β-lactamase gene *bla*_NDM-1_. Conjugation experiments showed that all three plasmids were capable of self-transfer *via* conjugation. Our results showed, for the first time, that this novel plasmid-mediated tigecycline resistance mechanism *TmexCD2-ToprJ2* has spread into different *Klebsiella* species, and clinical susceptibility testing may fail to detect. The co-occurrence of *bla*_NDM-1_ and *TmexCD2-ToprJ2* in the same plasmid is of particular public health concern as the convergence of “mosaic” plasmids can confer both tigecycline and carbapenem resistance. Its further spread into other clinical high-risk *Klebsiella* clones will likely exacerbate the antimicrobial resistance crisis. A close monitoring of the dissemination of *TmexCD-ToprJ* encoding resistance should be considered.

## Introduction

Tigecycline is one of the last-resort antibiotics used to treat severe infections caused by carbapenem-resistant Enterobacterales (Cheng et al., 2020). However, increasing studies reported the emergence of tigecycline resistance in clinical settings, which is frequently caused by the overexpression of non-specific active efflux pumps [*tet*(A) and *tet*(K)] or mutations within the drug-binding site in the ribosome [*tet*(M); Grossman, 2016; Linkevicius et al., 2016]. In *Klebsiella pneumoniae*, tigecycline resistance is also frequently associated with the overexpression of *ramA*, which can directly regulate multidrug resistance efflux pumps AcrAB and OqxAB (Ruzin et al., 2005, 2008). These tigecycline resistances are primarily mediated by chromosome-encoding mechanisms thus could not be easily transferred horizontally. The newly emerging mobile tigecycline resistance mechanism is of particular public health concern (He et al., 2019; Lv et al., 2020). The enzymatic modification gene variants [tet(X)] are highly transferable between species (Linkevicius et al., 2016; He et al., 2019; Sun et al., 2019).

Recently, a novel plasmid-mediated resistance-nodulation-division (RND)-type efflux pump gene cluster, *TmexCD1-ToprJ1*, has been identified in *K. pneumoniae* (Lv et al., 2020). This gene cluster was first identified in animal isolates but soon later was also found in clinical isolates (Sun et al., 2020). *TmexCD1-ToprJ1* was initially reported in China, but it has now been found in clinical *K. pneumoniae* isolates outside of China (Hirabayashi et al., 2021), suggesting this resistance has started to spread into other global regions. Two homologous variants, *TmexCD2-ToprJ2* (Wang et al., 2021a) and *TmexCD3-ToprJ3* (Wang et al., 2021b), have also been identified in *Raoultella ornithinolytica* and *Proteus mirabilis*, displaying similar tigecycline resistance profiles. Worrisomely, the mobile tigecycline gene clusters have also been found in clinical carbapenem-resistant *K. pneumoniae* strains (Chiu et al., 2017). Here, we reported the identifications of three clinical *TmexCD2-ToprJ2*-encoding Klebsiella strains, including two carbapenem-resistant strains co-harboring *bla*_VIM-8/NDM-5_ or *bla*_NDM-1_.

## Materials and Methods

### Bacterial Strains

*Klebsiella variicola* strain JNQH579 was recovered from a sputum sample of a 64-year male patient in intensive care units (ICUs) at a tertiary hospital in Jinan City, Shandong Province, in March 2021, who had been hospitalized for 17days due to severe pneumoniae and renal failure. The patient had received tigecycline treatment for 7days at a dosage of 100mg (IV) q12h before the isolation of the strain. Based on the antibiotic susceptibility testing results, antibiotic therapy was switched to aztreonam 0.5g (IV) q8h in combination with tigecycline. The respiratory symptoms improved after antibiotic treatment and the patient continued to be hospitalized for 3months due to cardiovascular and renal disease. *Klebsiella quasipneumoniae* strain JNQH473 was recovered from a urine sample of a 1-month-old infant with sepsis at a tertiary hospital in Xiamen City, Fujian Province, in August 2019, who was hospitalized at the department of pediatrics for 7days. During her hospitalization, the neonate received multiple antimicrobial treatments, including cefotaxime, vancomycin, and ceftazidime. The patient was fully recovered after antimicrobial and supportive treatment and was discharged on hospital day 13. *Klebsiella michiganensis* strain JNQH491 was recovered from a blood culture of a 48-year male patient at the department of oncology, who had received chemotherapy due to nasopharyngeal carcinoma in the same hospital as JNQH473. The patient was discharged home after a cycle of chemotherapy on hospital day 11. The overall strain features of the three strains are listed in Table 1. All the patients reported no recent travel abroad.

**Table 1:** Overall strain features of JNQH473, JNQH491, and JNQH579.

Characteristics	JNQH473	JNQH491	JNQH579
Species	*Klebsiella quasipneumoniae*	*Klebsiella michiganensis*	*Klebsiella variicola*
Isolation location	Xiamen	Xiamen	Jinan
Sector	Department of pediatrics	Department of oncology	Intensive care units
Host disease	Urinary infection, septic	Nasopharyngeal carcinoma	Pneumonia, renal failure
Isolation site	Urine	Blood	Sputum
Antimicrobial treatment before isolation	Cefotaxime, vancomycin, ceftazidime	–	Tigecycline
Collection time	2019.08	2020.08	2021.03
MLST	ST571	ST109	ST2013
K locus	KL64	ND	ND
O locus	O5	ND	OL103

MLST, multilocus sequence typing; K locus, *Klebsiella pneumoniae* species complex capsule locus; O locus, *K. pneumoniae* species complex LPS locus; ND, not determined.

### Antibiotic Susceptibility Testing

Antibiotic susceptibility testing (AST) was performed using the VITEK 2 (bioMérieux, Nürtingen, Germany) system. Minimum inhibitory concentrations (MICs) for tigecycline were performed by broth microdilution according to the Clinical and Laboratory Standards Institute (CLSI) guidelines. ATCC 25922 (*Escherichia coli*) and ATCC 27853 (*Pseudomonas aeruginosa*) were used as quality control strains for susceptibility testing. All the tests were performed in duplicate in different days. The breakpoints were interpreted according to CLSI guidelines except for tigecycline, of which the EUCAST epidemiological cutoff value >2μg/ml (for *K. pneumoniae*) was used.^1^

### Whole-Genome Sequencing, Assembly, and Annotation

The combination Oxford Nanopore (MinION system) and Illumina sequencing (NovaSeq) were used to achieve a high-quality genome assembly. First, we derived fastq read sequences from MinION raw electric signal fast5 files using guppy 3.2.2 with the high accuracy flip-flop algorithm. Adapters were trimmed out with Porechop.^2^ Low-quality reads were filtered out using trimmomatic 0.38 (Bolger et al., 2014). The filtered nanopore reads were *de novo* assembled with Flye 2.8.3 (Kolmogorov et al., 2019). Then, the obtained assemblies were polished using Nanopore reads by Racon 1.4.3 (1–4 iterations; Vaser et al., 2017). Next, the polished sequences were additionally corrected using Illumina reads by Pilon 1.23 until no changes occur (Walker et al., 2014). The whole-genome sequences were annotated by Prokka (Seemann, 2014) and RAST (Brettin et al., 2015), followed by manually curations.

### Genomic Analysis

The *in silico* multilocus sequence typing (MLST) was carried out using MLST v. 2.19.0,^3^ and antibiotic resistance/plasmid replicon gene detections were carried out using ABRicate v. 0.9.9^4^ using CARD (Jia et al., 2017) and PlasmidFinder (Carattoli et al., 2014) database, respectively. The intrinsic variants of *oqxAB*, chromosomal *ampH* and *fosA*, which confer resistance to quinolones, β-lactams and fosfomycin, respectively, in Enterobacterales rather than *Klebsiella* species, are therefore not reported (Henderson et al., 1997; Lam et al., 2021). Kleborate v. 1.0.0 (Lam et al., 2021) was used for *Klebsiella* K locus and O locus typing. The comparative analysis of *TmexCD2-ToprJ2* harboring plasmids was done by BLASTn and illustrated using CGView Server (Grant and Stothard, 2008). Easyfig (Sullivan et al., 2011) was used to visualize the genetic context comparisons. Selected plasmids were compared with Mauve 2.3.1 (Darling et al., 2010), followed by visualization using genoPlotR (Guy et al., 2010). Plasmid distance trees were generated using Mashtree (Katz et al., 2019). Alignment between three *TmexCD-ToprJ* variants was done by Clustal Omega (Madeira et al., 2019). ISFinder^5^ was used to identify ISs. Tn number was identified using Tn Number Registry (Roberts et al., 2008). In order to examine the distribution and relationships of *TmexCD2-ToprJ2* and its variants (*TmexCD1-ToprJ1*, *TmexCD2-ToprJ2*), plasmid sequences were downloaded from NCBI^6^ and compared.

### Conjugation Experiment

Conjugation experiments were performed by plate mating using *E. coli* J53 (AziR) as the recipient as described in our previous study (Hao et al., 2021). Briefly, overnight cultures of the donor strains and the recipient strain *E. coli* J53 were mixed (1:1) and applied onto an LB agar plate, followed by overnight culture at 37°C. Transconjugants were selected on Mueller-Hinton (MH) agar containing sodium azide (200μg/ml) and tigecycline (0.5μg/ml). The presence of *TmexCD2-ToprJ2* and IncHI1B replicons, as well as *bla*_NDM-1_ and *sul1* resistance genes in transconjugants, was confirmed by PCR (Supplementary Table S1). Conjugation frequency was calculated by dividing the number of transconjugants by the number of recipient cells. AST of the *E. coli* J53 transconjugants was performed as described above.

## Results

### Susceptibility of Three *Klebsiella* Isolates

The MICs for JNQH473, JNQH491, and JNQH579 were shown in Table 2. The MICs of tigecycline in JNQH473 and JNQH579 were both 4μg/ml. However, JNQH491 was susceptible to tigecycline with a MIC value of 1μg/ml. These strains were resistant to almost all tested β-lactam antibiotics except that JNQH491 and JNQH579 were susceptible to aztreonam. All the strains were susceptible to amikacin with an MIC value of less than 2μg/ml.

**Table 2:** Minimum inhibitory concentration (MIC) profiles of parental strains and trans-conjugants (μg/ml).

Strains	β-lactams									Aminoglycosides			Quinolones		Tetracycline	Others	
	AMP	AMC	PIPC/TAZ	FOX	CRO	FEP	ATM	ETP	IMP	AK	GEN	TOB	CIP	LEV	TIG	NFT	SMZ/TMP
JNQH473	≥32	≥32	≥128	≥64	≥64	≥64	16	≥8	≥16	≤2	≥16	8	2	1	4	128	≥320
JNQH491	≥32	≥32	≥128	≥64	≥64	16	≤1	≥8	8	≤2	≥16	2	1	1	1	64	≥320
JNQH579	≥32	≥32	≥128	≥64	≥64	≥64	≤1	≥8	≥16	≤2	≤1	8	2	1	4	32	≥320
*Escherichia coli* J53	8	4	<4	8	≤1	≤1	≤1	≤0.5	≤1	≤2	≤1	≤1	≤0.25	≤0.25	0.125	≤16	≤20
*E. coli* J53 transconjugants																	
pJNQH473-3	**≥32**	**16**	≤4	**32**	≤1	≤1	≤1	≤0.5	≤1	≤2	**≥16**	≤1	**≥4**	**4**	**1**	≤16	**≥320**
pJNQH491-2	**≥32**	**≥32**	**≥128**	**≥64**	**≥64**	**≥64**	≤1	**≥8**	**≥16**	≤2	**≥16**	≤1	**≥4**	**4**	**1**	≤16	**≥320**
pJNQH579-2	**≥32**	**≥32**	**≥128**	**≥64**	**≥64**	**≥64**	≤1	**≥8**	**≥16**	≤2	≤1	≤1	**≥4**	**4**	**1**	≤16	**≥320**

AMP, ampicillin; AMC, Amoxicillin-clavulanate; PIPC/TAZ, piperacillin/tazobactam; TIG, tigecycline; FOX, cefoxitin; FEP, cefepime; CIP, ciprofloxacin; CRO, ceftriaxone; ATM, aztreonam; IMP, imipenem; ETP, ertapenem; AK, amikacin; TOB, tobramycin; GEN, gentamycin; LEV, levofloxacin; NFT, nitrofurantoin, SMZ/TMP, sulfamethoxazole/trimethoprim. MICs of tigecycline were determined by microdilution and of other antibiotics were performed using VITEK2 system. All tests were performed in duplicate, and each test included three biological replicates. Changed MIC values in the *E. coli* J53 transconjugants relative to *E. coli* J53 itself were highlighted in bold.

### Characterization of Carbapenem-Resistant *Klebsiella variicola* Isolate JNQH473

Strain JNQH473 was classified as sequence type 571 (ST571) based on the *in silico* MLST, and it belonged to KL64 capsule and O5 lipopolysaccharide serotypes (Table 1). It harbored a 5.39-Mb chromosome and four plasmids, designated pJNQH473-1 (229.2-Kb), pJNQH473-2 (70.9-Kb), pJNQH473-3 (297.9-Kb), and pJNQH473-4 (46.1-Kb), respectively (Table 3). Two metallo-β-lactamase (*bla*_MBL_) genes, *bla*_IMP-8_ and *bla*_NDM-5_, were located on incompatible FIB type plasmid pJNQH473-1 and IncX3 type plasmid pJNQH473-4, respectively. *bla*_IMP-8_ was located in class 1 integron In*655* carrying the gene cassette of *bla*_IMP-8_-*aacA4* (Jiang et al., 2017), and *bla*_NDM-5_ was located in an ΔTn*125*-like region containing *ble*_MBL_ and IS*26* genes downstream and IS*3000*, IS*30*, and IS*5* family transposase genes upstream. The *TmexCD2-ToprJ2* gene cluster was located on an IncHI1B type plasmid, pJNQH473-3. In addition to *TmexCD2-ToprJ2* genes, nine antimicrobial resistance genes were found on the same plasmid, including two copies of sulfonamide resistance gene *sul1* and single copy of aminoglycoside resistance gene *aac(3)-IId* and *aadA16*, quinolone-resistant gene *qnrS1*, macrolides resistance gene *mphA*, trimethoprim resistance gene *dfrA27*, the β-lactamase gene *bla*_TEM-1D_, and rifampicin resistance ribosyltransferase gene *arr-3*.

**Table 3:** Chromosome and plasmid features of strains in this study.

Strains	Chromosome or plasmid	Size (bp)	Plasmid type	Acquired AMR genes
*K. quasipneumoniae* JNQH473	Chromosome	5,390,886	–	*bla*_OKP-A-3_
	pJNQH473-1	229,265	IncFIB	*aac(6')-Ib4,bla*_CTX-M-14_,*bla*_IMP-8_
	pJNQH473-2	70,940	FII(pBK30683)	–
	pJNQH473-3^*^	297,981	IncHI1B	*aac(3)-IId;aadA16,qnrS1,mphA,arr-3,sul1,tmexCD2-toprJ2,dfrA27,bla*_TEM-1D_
	pJNQH473-4	46,166	IncX3_1	*bla*_NDM-5_
*K. michiganensis* JNQH491	Chromosome	6,039,729	–	*bla*_OXY-1-3_
	pJNQH491-1	205,582	IncFIB	–
	pJNQH491-2^*^	307,464	IncHI1B	*aac(3)-Iid,qnrS1,mphA,sul1,tmexCD2-toprJ2,dfrB4,bla*_TEM-1D_, *bla*_NDM-1_
*K. variicola* JNQH579	Chromosome	5,583,188	–	*bla*_OXY-1-3_
	pJNQH579-1	197,434	IncFIB	–
	pJNQH579-2^*^	369,339	IncHI1B	*aac(6')-Ib,aadA16,qnrS1,mphE,msrE,catB3,catII.2,arr-3,sul1,tmexCD2-toprJ2,dfrA27,bla*_OXA-1_,*bla*_NDM-1_

* *TmexCD2-ToprJ2* harboring plasmid.

### Characterization of Carbapenem-Resistant *Klebsiella michiganensis* Isolate JNQH491

**Figure 1:**
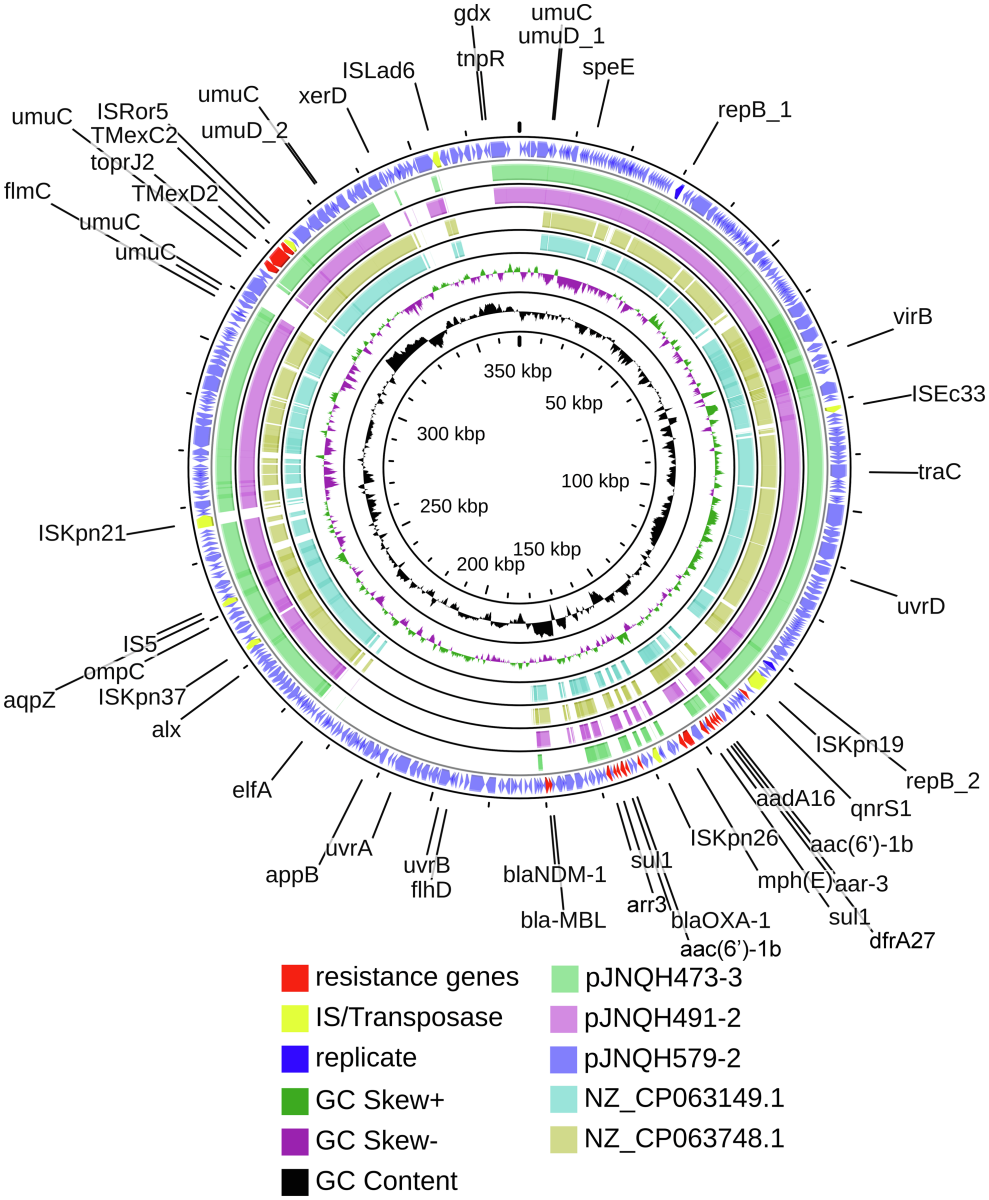
Comparative structural analysis of pJNQH473-3, pJNQH491-2, pJNQH579-2, and two *TmexCD2-ToprJ2* harboring plasmids NZ_CP063149.1 and NZ_CP063748.1. Open reading frames (ORFs) of pJNQH579-2 are shown as the outermost ring, with plasmid replicons, insertion sequences (IS), and antimicrobial resistance genes highlighted.

Strain JNQH491 belonged to ST109 type (Table 1). The K and O locus could not be classified according to the currently available K and O loci database. The genome contained a 6.04-Mb chromosome and two plasmids, pJNQH491-1 (205.5-Kb) and pJNQH491-2 (307.4-Kb; Table 3). The *TmexCD2-ToprJ2* gene cluster was located on pJNQH491-2, which is an IncHI1B type plasmid, co-harboring multiple resistant genes, including the *bla*_MBL_ and *bla*_NDM-1_ (Figure 1). The pJNQH491-1 plasmid had an IncFIB replicon and did not carry any known resistance genes.

### Characterization of Carbapenem-Resistant *Klebsiella quasipneumoniae* Isolate JNQH579

**Figure 2:**
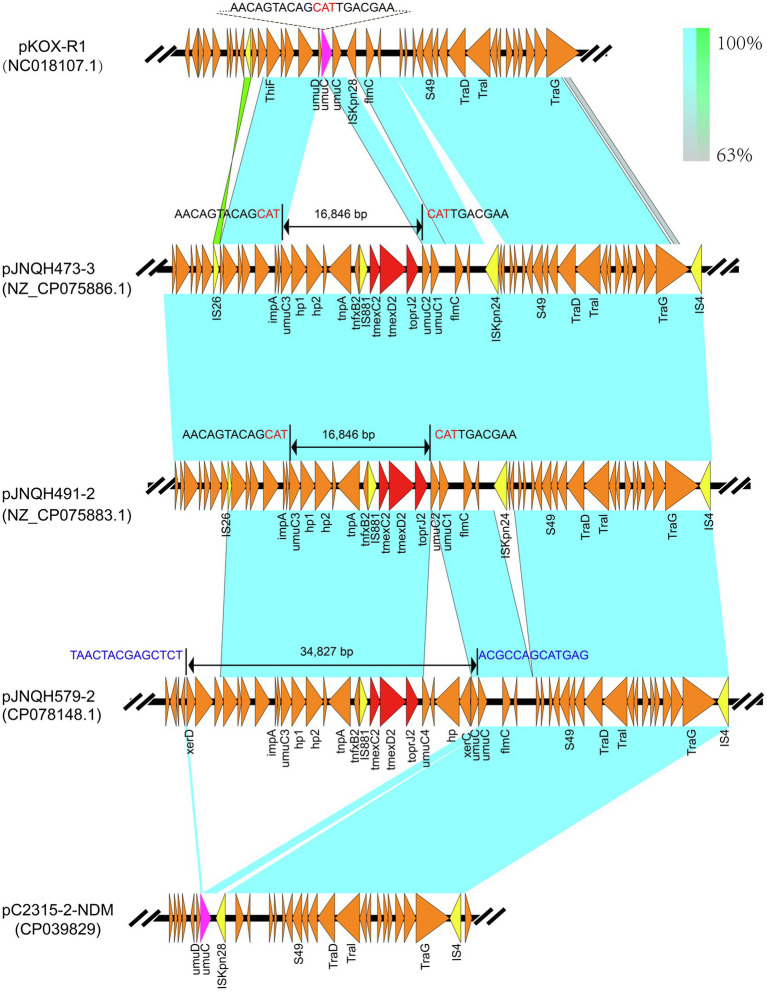
Linear comparisons of *TmexCD2-ToprJ2*-bearing genetic contexts in pJNQH473-3 and that in pJNQH579-2, pC2315-2-NDM, and plasmid pKOX_R1(NC018107.1). Light blue shading indicates shared regions of homology, while green shading indicates inversely displayed regions of homology. Colored arrows indicate ORFs. The red arrows indicate the antibiotic resistance genes. The yellow arrows indicate IS. The nucleotide sequence of the *umuC* gene (green arrow) representing the insertion site is shown above the gene. The 16,846bp putative “*hp1-hp2-tnpA-tnfxB2-IS881-TmexCD2-ToprJ2*” transposon located on pJNQH473-3 and 34,827bp larger putative transposon on pJNQH579-2 are marked with bilateral black arrows.

**Figure 3:**
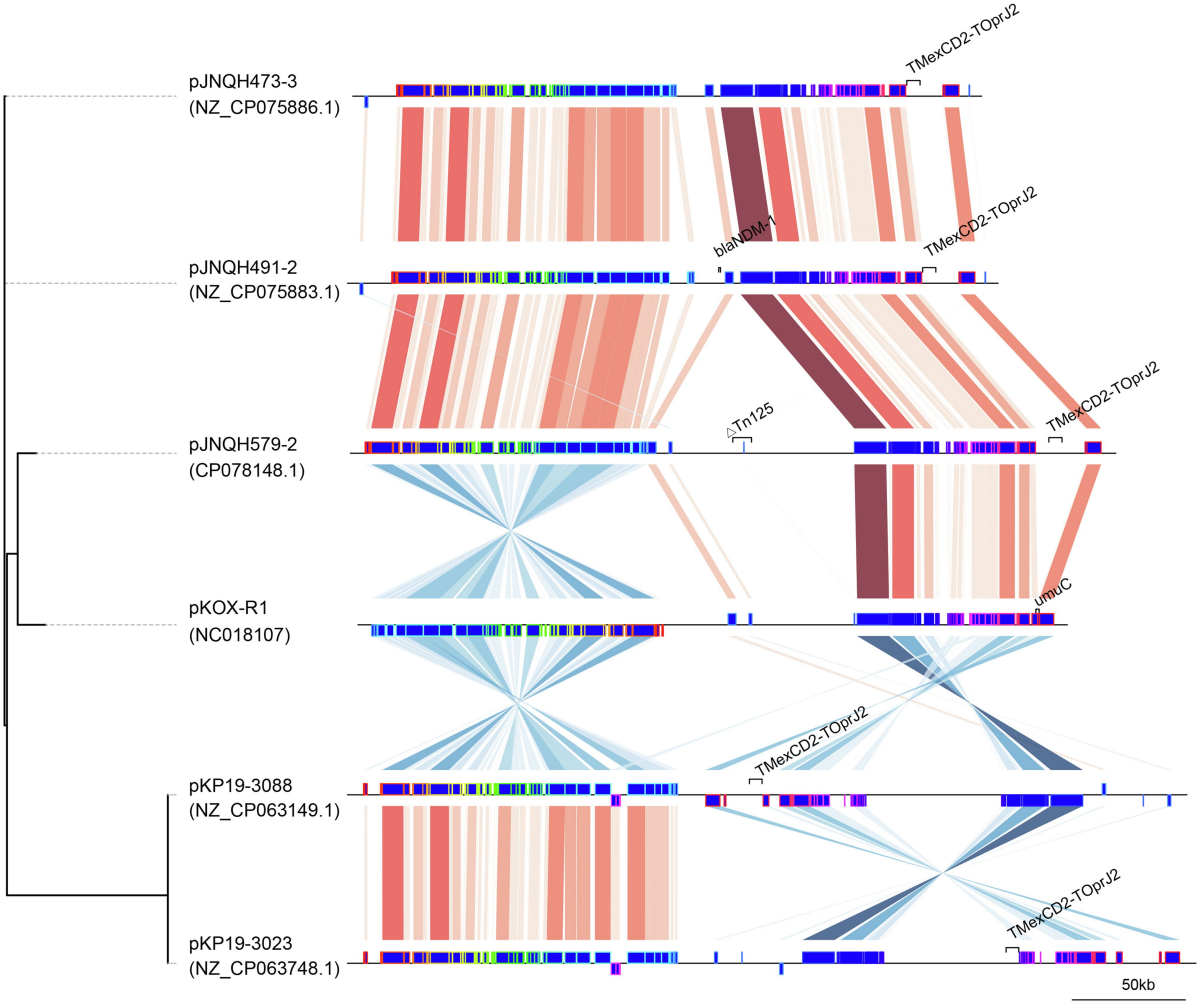
Comparison of linear plasmid maps of four *TmexCD2-ToprJ2*-bearing plasmids and the related plasmid pKOX-R1. The genomes were compared with Mauve and the elements were designated as Mauve blocks. Red shaded regions between plasmids indicates shared regions of homology, while blue shading indicates inversely displayed regions of homology. The tree was created using Mashtree.

Strain JNQH579 belonged to ST2013 and harbored an OL103 serotype (Table 1). The genome included a 5.58-Mb chromosome and two plasmids, designated pJNQH579-1 (197.4-Kb) and pJNQH579-2 (369.3-Kb), respectively (Table 3). The *TmexCD2-ToprJ2* gene cluster was carried by a 34,827bp mobile region, which was located on pJNQH579-2 plasmid (Figure 2). pJNQH579-2 is also an IncHI1B type plasmid, co-harboring multiple resistance genes [*aac(6′)-Ib*, *aadA16*, *qnrS1*, *mphE*, *msrE*, *catB3*, *catII.2*, *arr-3*, *sul1*, *dfrA27*, *bla*_OXA-1_, and *bla*_NDM-1_; Table 3]. Notably, the *bla*_NDM-1_ carbapenem-resistant gene was co-existed with *TmexCD2-ToprJ2* in pJNQH579-2 plasmid. *bla*_NDM-1_ was found within a truncated transposon Tn*125* (Figure 3), with the structure of “Δ*ISAba125-bla*_NDM-1_*-ble*_MBL_*-tat-dvt-groESL- tnpAISCR21*” (Poirel et al., 2012). The pJNQH579-1 plasmid had an IncFIB replicon but did not carry any known resistance genes.

### Comparative Genomic Analysis of HI1B Plasmids Carrying *TmexCD2-ToprJ2*

The *TmexCD2-ToprJ2* cluster in the three strains had almost 100% nucleotide identities to the cluster of the originally reported *TmexCD2-ToprJ2* (Wang et al., 2021a). Plasmid sequence comparison demonstrated that pJNQH473-3 and pJNQH491-2 had highly conserved plasmid synteny and structure, with over 99.9% nucleotide identities (Figure 3). pJNQH491-2 had a blast query coverage of 74% and over 99% nucleotide identities with pJNQH579-2. Further plasmid BLAST query against GenBank database showed that pJNQH473-3 and pJNQH491-2 were closely related to plasmid pKOX-R1 (NC018107), isolated from Taiwan in *K. michiganensis* E718 (Figure 3). In comparison with pKOX-R1, a 16,846bp region, containing the *TmexCD2-ToprJ2*, was inserted into the *umuC* gene of pJNQH473-3, generated a 3bp (CAT) direct repeats (Figure 2). The three pJNQH plasmids contain the same *TmexCD2-ToprJ2* structure of “*hp1-hp2-tnpA-tnfxB2-IS881-TmexCD2-ToprJ2*,” with an average GC content of 42.3%. pJNQH579-2 was found to be closely related to plasmid pC2315-2-NDM (CP039829). The putative “*hp1-hp2-tnpA-tnfxB2-IS881-TmexCD2-ToprJ2*” transposon was surrounded by *XerC, XerD*, and additional hypothetical genes. This region was also inserted into the *umuC* gene therefore constituted a putative larger transposon unit of 34,827bp (Figure 2).

### Sequence Comparison of Three *TmexCD-ToprJ* Gene Clusters

*TmexCD2-ToprJ2* cluster shares a high similarity to *TmexCD1-ToprJ1*, of which the variants of *TmexC*, *TmexD*, and *ToprJ* genes had 98.02, 96.75, and 99.93% nucleotide identities and 97.67, 97.61, and 99.79% amino acid identities between each other. Compared with *TmexCD3-ToprJ3*, the variants of *TmexC*, *TmexD*, and *ToprJ* genes shared 94, 96.72, and 99.86% nucleotide identities and 97.12, 98.08, and 99.79% amino acid identities. *ToprJ* gene shares the highest similarity at the amino acid level among the three variants, of which *ToprJ2* differs from *ToprJ1* and *ToprJ3* by a single amino acid substitution (Ala47Thr; Supplementary Figure S1).

### Genomic Analysis of Plasmids Harboring *TmexCD-ToprJ* Variants

**Figure 4:**
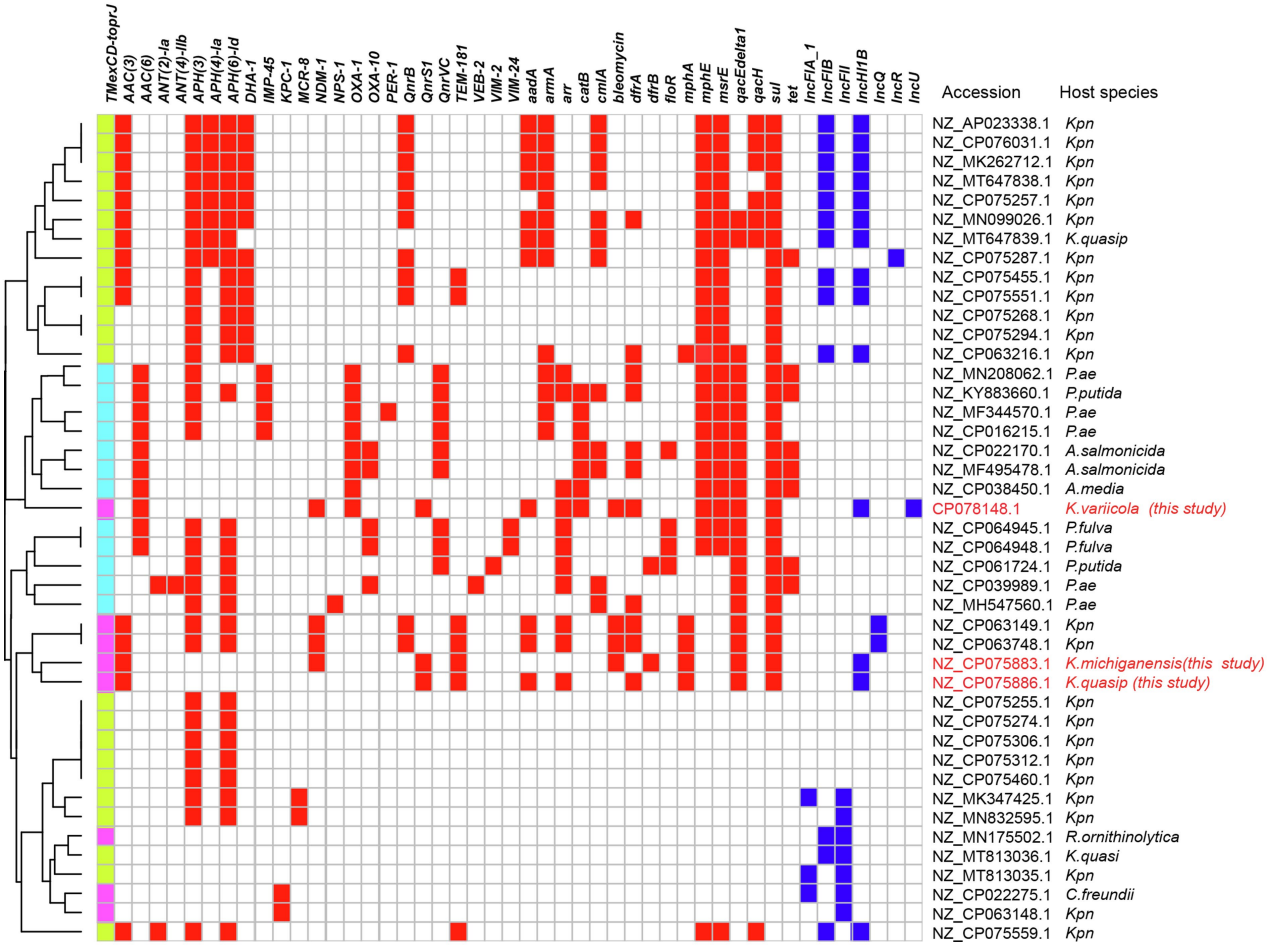
*TMexCD-toprJ*-harboring plasmids. The heatmap shows the distribution of plasmid replicons (dark blue boxes) and antibiotic resistance genes (red boxes) detected within 51 *TMexCD-toprJ*-harboring plasmids. The variants of *TMexCD-toprJ* resistance genes are indicated in green (*TMexCD1-toprJ1*), pink (*TMexCD2-toprJ2*), and light blue (*TMexCD3-toprJ3*) boxes. GenBank accession numbers and species are listed on the right-hand side. *Kpn*, *Klebsiella pneumoniae*; *K. quasip*, *Klebsiella quasipneumoniae*; *Pae*, *Pseudomonas aeruginosa*; *P. putida*, *Pseudomonas putida*; *A. salmonicida*, *Aeromonas salmonicida*; *A. media*, *Aeromonas media*; *K. variicola*, *Klebsiella variicola*; *P. fulva*, *Pseudomonas fulva*; *P.* sp.*, Pseudomonas* species; *R. ornithinolytica*, *Raoultella ornithinolytica*; *C. freundii*, *Citrobacter freundii*.

The analysis of plasmid database revealed that a total of 43 plasmids carried *TMexCD1-ToprJ1* (*n*=23), *TmexCD2-ToprJ2* (*n*=8), and *TmexCD3-ToprJ3* (*n*=12) gene clusters (with 100% gene coverage and over 99.97% identity), which were distributed mainly in *Enterobacteriaceae* and *P. aeruginosa* (Figure 4). *TmexCD1-ToprJ1* and *TmexCD2-ToprJ2* were mostly identified in *K. pneumoniae*, whereas *TmexCD3-ToprJ3* was more frequently found in *P. aeruginosa*. In addition to *Klebsiella*, the *TmexCD2-ToprJ2* harboring plasmids were also found in *R. ornithinolytica* (*n*=1), *Citrobacter freundii* (*n*=1). Most *TmexCD2-toprJ2* gene clusters were associated with IncFII (*n*=3), IncHI1B (*n*=3), and IncQ (*n*=2) plasmids. Of note, six *TmexCD2-ToprJ2* harboring plasmids also co-harbored carbapenem-resistant genes, including four *bla*_NDM-1_ and two *bla*_KPC-1_ genes.

### Transfer of *TmexCD2-ToprJ2* Harboring Plasmids *via* Conjugation

The *TmexCD2-ToprJ2* harboring IncHI1B plasmids were successfully transferred into *E. coli* J53 from three JNQH strains. Further PCRs confirmed that other plasmids in the donor strains were not co-transferred along with the *TmexCD2-ToprJ2* harboring IncHI1B plasmids into the recipient *E. coli* J53 strain (Supplementary Figure S2). The conjugation frequency was 10^−6^, 10^−6^, and 10^−5^ for JNQH473, JNQH491, and JNQH579 per recipient cell, respectively. The MICs for the *E.coli* J53 transconjugants are shown in Table 2. The MICs of tigecycline against *E. coli* J53 transconjugants were 1μg/ml, which were 8-fold higher than that of the *E. coli* J53 itself. In addition, the transfer of *bla*_NDM-1_ together with the *TmexCD2-ToprJ2* genes for JNQH491 and JNQH579 in the *E. coli* J53 transconjugants conferred resistance to all tested β-lactams (ampicillin, amoxicillin-clavulanate, piperacillin/tazobactam, cefoxitin, ceftriaxone, cefepime, imipenem, and ertapenem) except for aztreonam. All transconjugants exhibited resistance to quinolones (ciprofloxacin and levofloxacin, ≥4μg/ml), which was likely due to the combinational effects of both quinolone resistance determinant *qnrS1* and the *TmexCD2-ToprJ2*. Further, *E.coli* J53 transconjugants for JNQH491 and JNQH579 exhibited resistance to gentamycin, while it was susceptible for JNQH473. *Aac(3)-IId* is likely the main source of the disparity on the basis that *aac(3)-IId* was co-harbored with *TmexCD2-ToprJ2* in the same plasmids for JNQH473 and JNQH491 strains, while it was absent on the pJNQH579-2 plasmid.

## Discussion

*TmexCD1-ToprJ1* is the first reported plasmid-encoded RND efflux pump, conferring resistance to multiple drugs including tigecycline (Lv et al., 2020). More recently, a variant of *TmexCD2-ToprJ2* was identified in a clinical *R. ornithinolytica* strain NC189, which demonstrated similar tigecycline resistance as the *TmexCD1-ToprJ1* (Wang et al., 2021a). In comparison with *TmexCD1-ToprJ1*, most of which were detected in *K. pneumoniae* (Figure 2), *TmexCD2-ToprJ2* was identified among a variety of *Enterobacteriaceae* species, including *R. ornithinolytica*, *C. freundii*, *Aeromonas hydrophila*, and *K. pneumoniae* (Wang et al., 2021a). Our study demonstrated that the *TmexCD2-ToprJ2* had spread into carbapenem-resistant *K. pneumoniae* species complex (KpSC), which is an emerging pathogenic species and frequently detected clinically (Wyres et al., 2020). The highly conserved synteny and structure of the *TmexCD2-ToprJ2* harboring plasmids suggested the likelihood of horizontal transfer of a highly similar plasmid between different *Klebsiella* species. We also found the co-existence of *TmexCD2-ToprJ2* and *bla*_NDM-1_ genes in pJNQH491-2 and pJNQH579-2, confirmed by the resistance profile of the transconjugants. The co-transfer of *bla*_NDM-1_ with *TmexCD2-ToprJ2* for JNQH491 and JNQH579 in the *E. coli* J53 conjugants conferred resistance to tigecycline and all tested β-lactams except aztreonam. In addition, analysis of plasmids from the database from NCBI revealed frequent co-occurrence of *TmexCD2-ToprJ2* and carbapenem-resistant genes in the same plasmid. Our finding is of particular public health concern as the convergence of “mosaic” plasmids can confer both tigecycline and carbapenem resistance, thus leading to a serious challenge to the treatment of bacterial infections.

It has been demonstrated in a previous study that *TmexCD2-ToprJ2* functions as an efflux pump system by the efflux inhibition experiments (Wang et al., 2021a). *TmexCD2-ToprJ2* exhibits a broad substrate spectrum toward tetracyclines, eravacycline, tigecycline (8-fold MIC increase), ciprofloxacin (4-fold MIC increase), and slightly decreased susceptibility (2-fold MIC increase) to cefotaxime and cefepime (Wang et al., 2021a). Our finding is consistent with previous study that *TmexCD2-ToprJ2* gene cluster caused 8-fold increase in the tigecycline MICs in the *E. coli* transconjugants (Wang et al., 2021a). However, strain *K. michiganensis* JNQH491 was susceptible to tigecycline with a MIC value of 1μg/ml irrespective of the presence of *TmexCD2-ToprJ2* gene. We speculated that the differences in plasmid copy numbers or transcription of promoter sequence in this *K. michiganensis* strain might contribute to the different susceptibility profiles observed in strains from this study (Shaheen et al., 2011). Further studies are needed to evaluate how much *TmexCD2-ToprJ2* will affect the therapeutic effects of tigecycline *in vivo*, including clinical isolates with low MIC values.

An increasing number of studies have supported that the *TmexCD-ToprJ*-like efflux pump system may originate from the chromosome of *Pseudomonas* species, as their structures are closely related to the chromosomal *MexCD-OprJ* system in *P. aeruginosa* (Lv et al., 2020; Sun et al., 2020; Wang et al., 2021a,b). These findings suggested *TmexCD-ToprJ*-like clusters might be originated from chromosomal genes in *Pseudomonas* species, through horizontal transfer into *Enterobacteriaceae* species. The *MexCD-OprJ* family proteins act as efflux pumps, conferring intrinsic resistance to tetracycline, chloramphenicol, and norfloxacin in *P. aeruginosa* (Poole et al., 1993). The rapid expansion of the *TmexCD-ToprJ* cluster has been attributed to various mobile genetic elements, such as ICEs, transposons (e.g., Tn5393), or IS element (e.g., IS*26*; Sun et al., 2020; Wan et al., 2020; Wang et al., 2021b; Yang et al., 2021). In our study, a genetic structure (*hp1-hp2-tnpA-tnfxB2-IS881-TmexCD2-ToprJ2*) constitutes a putative transposon system. Further, a larger putative transposon comprised of 34,827bp harboring *TmexCD2-ToprJ2* was also inserted into the *umuC*-like gene of pJNQH579-2. As such, the *umuC* gene appears to be a “hotspot” for *TmexCD2-ToprJ2* integration in IncHIB plasmids, while the molecular mechanism underlying the site-specific integration deserves further studies.

## Conclusion

Overall, we report the first time of three IncHI1B type plasmids encoding efflux pump *TmexCD2-ToprJ2* in carbapenem-resistant *K. variicola*, *K. michiganensis*, and *K. quasipneumoniae* species. The sequence analysis identified a putative transposon element for *TmexCD2-ToprJ2* transmission, with the genetic structure of “*hp1-hp2-tnpA-tnfxB2-IS881-TmexCD2-ToprJ2*.” Of note, the co-existence of *bla*_NDM-1_ and *TmexCD2-ToprJ2* is of particular public health concern. The emergence of such *Klebsiella* strains underscores the importance of clinical awareness of this pathotype and the need for continued monitoring of *TmexCD-ToprJ* family resistance genes in China and around the world.

## Data Availability Statement

Complete sequences of the chromosomes and plasmids of strain JNQH473, JNQH491, and JNQH579 have been deposited in the GenBank databases under accession numbers NZ_CP075884.1-NZ_CP075888.1, NZ_CP075881.1-NZ_CP075883.1, and NZ_CP078146.1-NZ_CP078148.1, respectively.

## Ethics Statement

The studies involving human participants were reviewed and approved by the First Affiliated Hospital of Shandong First Medical University. Written informed consent to participate in this study was provided by the participants’ legal guardian/next of kin. Written informed consent was obtained from the individual(s), and minor(s)’ legal guardian/next of kin, for the publication of any potentially identifiable images or data included in this article.

## Author Contributions

MH and WM conceived the study and designed the experimental procedures. BZ and XD collected the strains. YW, BZ, ML, JM, XL, and FC performed the experiments. MH, JG, and SL analyzed the data. WM, YH, and YW contributed to reagents and materials. MH and LC wrote the manuscript. All authors contributed to the article and approved the submitted version.

## Conflict of Interest

The authors declare that the research was conducted in the absence of any commercial or financial relationships that could be construed as a potential conflict of interest.

## Publisher’s Note

All claims expressed in this article are solely those of the authors and do not necessarily represent those of their affiliated organizations, or those of the publisher, the editors and the reviewers. Any product that may be evaluated in this article, or claim that may be made by its manufacturer, is not guaranteed or endorsed by the publisher.

## Footnotes

^1^ https://eucast.org/clinical_breakpoints/

^2^ https://github.com/rrwick/Porechop

^3^ https://github.com/tseemann/mlst

^4^ https://github.com/tseemann/abricate

^5^ https://isfinder.biotoul.fr/

^6^https://ftp.ncbi.nlm.nih.gov/refseq/release/plasmid, latest update on July 15, 2021.

## Supplementary Material

Supplementary Figure S1 Multiple sequence alignment of amino acids of *TmexCD1-ToprJ1*, *TmexCD2-ToprJ2*, and *TmexCD3-ToprJ3*. Conserved residues were displayed in dark blue background and mutants were highlighted in white background.

Supplementary Figure S2 PCR amplification of plasmid replicons and resistance genes of JNQH473, 491, 579, and their *E. coli* J53 transconjugants.
