# Supplementary figures and images for: Characterization of IncHI1B Plasmids Encoding Efflux Pump TmexCD2-ToprJ2 in Carbapenem-Resistant Klebsiella variicola, Klebsiella quasipneumoniae, and Klebsiella michiganensis Strains
Source: Front Microbiol. 2021 Oct 6;12:759208. doi: 10.3389/fmicb.2021.759208 (PMC8527040; doi:10.3389/fmicb.2021.759208)

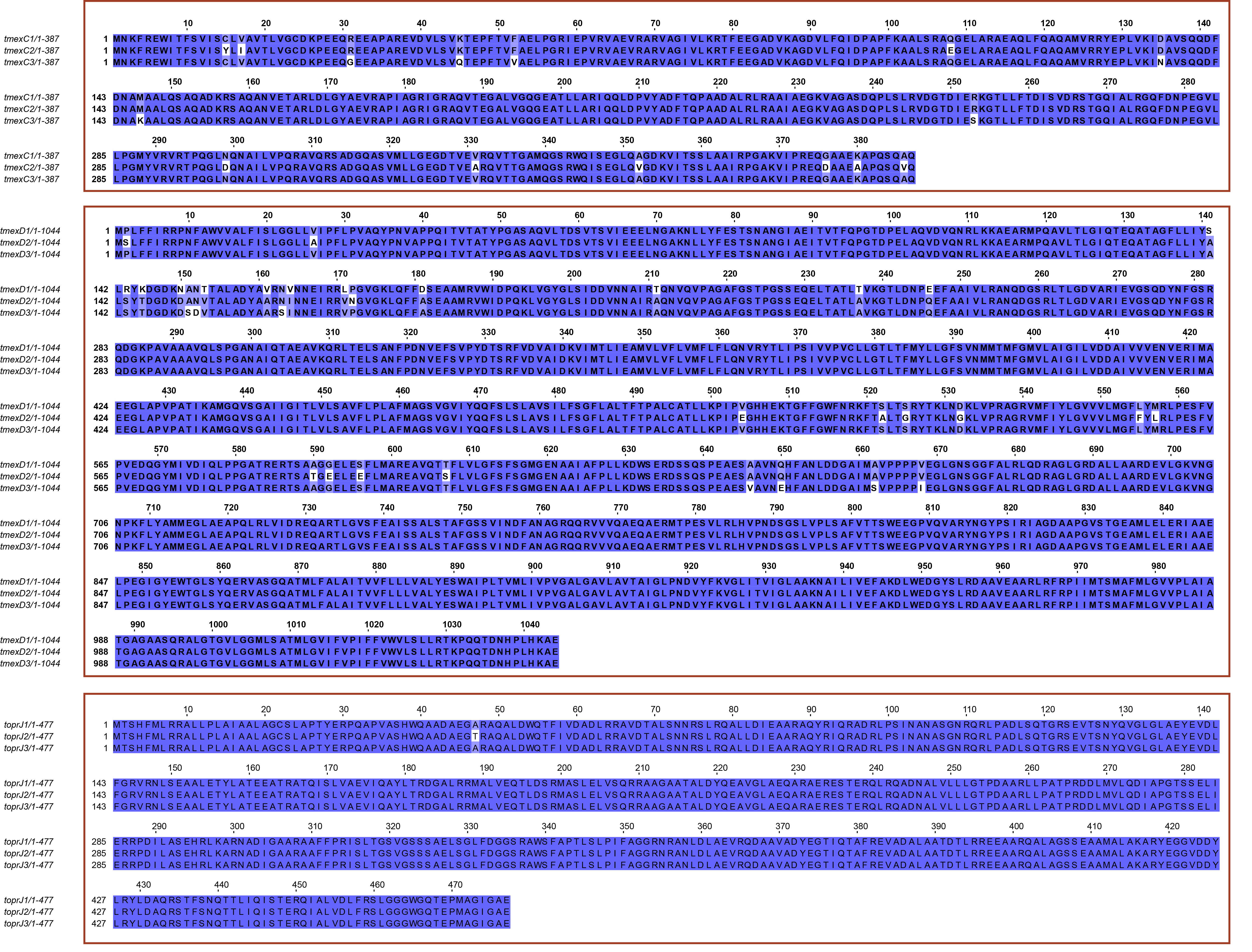

Supplement: Supplementary Figure S1 — Multiple sequence alignment of amino acids of TmexCD1-ToprJ1, TmexCD2-ToprJ2, and TmexCD3-ToprJ3. Conserved residues were displayed in dark blue background and mutants were highlighted in white background. [file Image_1.TIF]

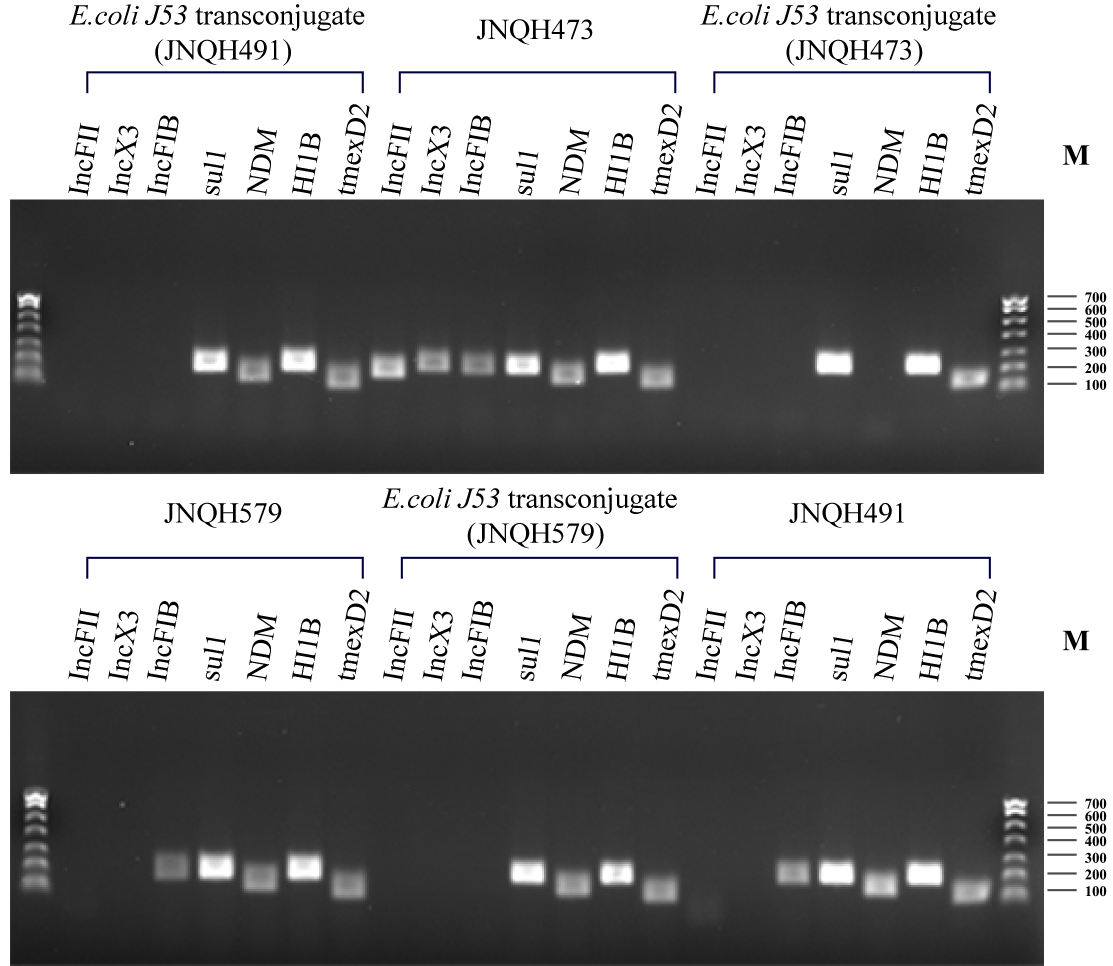

Supplement: Supplementary Figure S2 — PCR amplification of plasmid replicons and resistance genes of JNQH473, 491, 579, and their E. coli J53 transconjugants. [file Image_2.TIF]
