# Supplementary material for: Characterization of IncHI1B Plasmids Encoding Efflux Pump TmexCD2-ToprJ2 in Carbapenem-Resistant Klebsiella variicola, Klebsiella quasipneumoniae, and Klebsiella michiganensis Strains
Source: Front Microbiol. 2021 Oct 6;12:759208. doi: 10.3389/fmicb.2021.759208 (PMC8527040; doi:10.3389/fmicb.2021.759208)
Supplement: Supplementary file 3 [file Table_1.DOCX]

**Supplementary Table 1. Primers used in this study**

| **Target gene** | **Name** | **Sequence** | **Product size** |
| --- | --- | --- | --- |
| *tmexD2* | tmexD2_F | 5’-AGATCACCGTCACCTTCCAG-3’ | 159 |
|  | tmexD2_R | 5’-GCGCATAGATCAGCAGGAAG-3’ |  |
| *IncHI1B* | IncHI1B_F | 5’-ACACCGATTTTCGAGTCTGG-3’ | 246 |
|  | IncHI1B_R | 5’-AACGTGCCCAGTTTATCAGG-3’ |  |
| *bla*_NDM_ | NDM_F | 5’-GATTGCGACTTATGCCAATG-3’ | 189 |
|  | NDM_R | 5’-TCGATCCCAACGGTGATATT-3’ |  |
| *Sul1* | Sul1_F | 5’-GACTGCAGGCTGGTGGTTAT-3’ | 244 |
|  | Sul1_R | 5’-CCGACTTCAGCTTTTGAAGG-3’ |  |
| *IncFIB* replicon | IncFIB-F | 5’-TTTGGTCTCGCTCTGGAACT-3’ | 239 |
|  | IncFIB-R | 5’-ACGTTAAGATCACCGGTTCG-3’ |  |
| *IncX3* replicon | IncX3-F | 5’-TTGGGGTAACTCTTGCATCC-3’ | 248 |
|  | IncX3-R | 5’-CCCAAGTTCATCAGCCAAAT-3’ |  |
| *IncFII* replicon | IncFII-F | 5’-CAATATGGCCATCGAGTGTG-3’ | 213 |
|  | IncFII-R | 5’-ACCACAGCCACCTCAGAAAC-3’ |  |
